# Fast identification of the conduction-type of nanomaterials by field emission technique

**DOI:** 10.1038/s41598-017-12741-5

**Published:** 2017-10-12

**Authors:** Xun Yang, Haibo Gan, Yan Tian, Luxi Peng, Ningsheng Xu, Jun Chen, Huanjun Chen, Shaozhi Deng, Shi-Dong Liang, Fei Liu

**Affiliations:** State Key Laboratory of Optoelectronic Materials and Technologies, Guangdong Province Key Laboratory of Display Material and Technology, School of Electronics and Information Technology, Sun Yat-sen University, Guangzhou, 510275 P. R. China

## Abstract

There are more or less dopants or defects existing in nanomaterials, so they usually have different conduct-types even for the same substrate. Therefore, fast identification of the conduction-type of nanomaterials is very essential for their practical application in functional nanodevices. Here we use the field emission (FE) technique to research nanomaterials and establish a generalized Schottky-Nordheim (SN) model, in which an important parameter λ (the image potential factor) is first introduced to describe the effective image potential. By regarding λ as the criterion, their energy-band structure can be identified: (a) λ = 1: metal; (b) 0.5 < λ < 1: n-type semiconductor; (c) 0 < λ < 0.5: p-type semiconductor. Moreover, this method can be utilized to qualitatively evaluate the doping-degree for a given semiconductor. We test numerically and experimentally a group of nanomaterial emitters and all results agree with our theoretical results very well, which suggests that our method based on FE measurements should be an ideal and powerful tool to fast ascertain the conduction-type of nanomaterials.

## Introduction

With the rapid development of IT industry, more and more device integration is intensively demanded, which inversely drives the scientists and researchers all over the world to explore new approaches to break through the size limit of traditional semiconductor devices. Under this circumstance, nanodevices have been believed as the necessary choices for meeting their increase requirements. Quasi one-dimensional nanomaterials (nanowires, nanorods, etc.) are the ideal building blocks for fabrication of nanodevices because of their distinctive and excellent properties, such as Si nanowires^1–4^ and ZnO nanowires^5–7^. Up to date, various preparation methods^8–13^ have been developed to synthesize different nanomaterials, but there inevitably exist some impurity dopants or defects in their formation process. As a result, nanomaterials by different preparation ways usually exhibit diverse conduction-type in comparison with their perfect single crystal counterparts. Therefore, accurately differentiating the conduction-type of nanomaterials is of great significance for nanodevices because their conduction-type directly determines their application area (photovoltaic, memory, switching or electron-source devices).

As known to all, there are two commonly-used methods for identification of the conduction type of bulk or thin film materials, which are respectively the hot and cold probe method and Hall effect method^14^. The former utilizes the thermoelectric effect of bulk materials while the working mechanism of the latter is based on the calculation of the steady electric potential difference between the top and down surface of the sample resulting from the carrier charges under electromagnetic field. But for nanomaterials, integrating them into field effect transistor (FET) and measuring their working behaviors is the only known method to identify their conduction-type to our knowledge ^15,16^. Although this method has a nice measurement accuracy, the fabrication process of FET nanodevices is quite complicated and the yield of nanodevices with nice working performances is usually rather low. So finding an easy and accurate method to differentiate the conduction-type of nanomaterials becomes a challenging issue for all the researchers.

In this paper, we will apply the simple FE techniques to identify the conduction-type of quasi one-dimensional nanomaterials. Based on the FE experiments, we further establish a generalized Schottky-Nordheim (SN) field emission model, in which an important factor will be first introduced to describe the effective image potential in Sec. II. Also, we will present a programmed scheme to fit the J-E curves and the slope of FN plots by introducing several parameters, by which the connection between the important factor and the energy-band structure of nanomaterials can be obtained in Sec. III. The numerical and experimental testing will be presented in Sec. IV. Finally, we give some detailed discussions and conclusions.

## Generalized Schottky-Nordheim model

**Figure 1:**
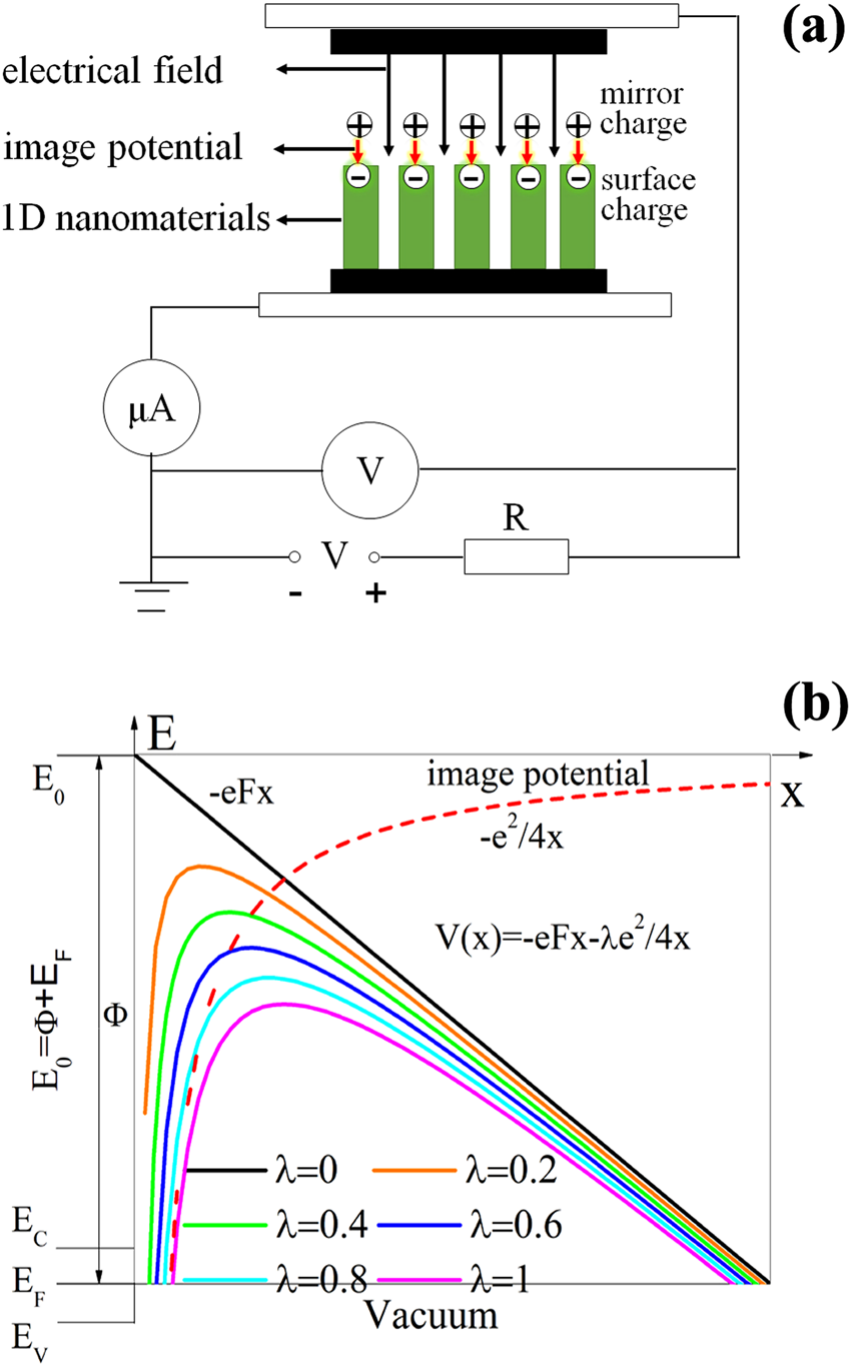
(**a**) The schematic diagram of field emission measurement. (**b**) The generalized Schottky-Nordheim (SN) model.

The current-voltage (I-V) curve plays a crucial role in understanding the basic physics of field electron emission. The first theoretical field emission (FE) model was proposed by Fowler-Nordheim (FN) based on Sommerfeld model with some approximations, which was widely used in analyzing the FE behaviors of metallic emitters. Subsequently, it was further modified by Schottky-Nordheim (SN), in which the image potential is taken into account according to electrodynamics^17^. It is known that both of FN and SN models are assumed that the emitters are metallic. However, recently many new-type nanomaterials are applied in FE process. The schematic diagram of quasi one-dimensional nanomaterials during FE measurements is provided in Fig. 1(a). In FE process, the electrons emit from the cathode sample surface at negatively biased. The emergence of image potential at boundary will lower the surface barrier height, which results that the electron emission is easy to occur at applied field, as seen in Fig. 1(b). Commonly, the energy-band structures of nanomaterials are quite different from those of metals. As a result, the image potential effects will differ with the energy-band structures of nanomaterials because their electric conductivity is various in the band diagram.

In order to analyze the FE behaviors from nanomaterials, we first introduce a parameter λ (0 < λ < 1) to generalize the SN model, in which λ is defined as the image potential factor. So the current density *J*_*GSN*_ can be rewritten as

1 $$\begin{array}{rcl}{J}_{GSN} & = & a{{\tau }_{F}}^{-2}\frac{{F}^{2}}{\Phi }exp(-{\nu }_{F}\frac{b{\Phi }^{3/2}}{F}){\Theta }_{GSN}(T)\\  & = & a{(1+\frac{1}{9}\lambda \frac{cF}{{\Phi }^{2}}-\frac{1}{18}\lambda \frac{cF}{{\Phi }^{2}}ln\lambda \frac{cF}{{\Phi }^{2}})}^{-2}\frac{{F}^{2}}{\Phi }exp\\  &  & \times (-(1-\lambda \frac{cF}{{\Phi }^{2}}+\,\frac{1}{6}\lambda \frac{cF}{{\Phi }^{2}}ln\lambda \frac{cF}{{\Phi }^{2}})\frac{b{\Phi }^{3/2}}{F})\\  &  & \times \frac{(1+\frac{1}{9}\lambda \frac{cF}{{\Phi }^{2}}-\frac{1}{18}\lambda \frac{cF}{{\Phi }^{2}}ln\lambda \frac{cF}{{\Phi }^{2}}){k}_{B}T\pi /{d}_{F}}{\sin ((1+\frac{1}{9}\lambda \frac{cF}{{\Phi }^{2}}-\frac{1}{18}\lambda \frac{cF}{{\Phi }^{2}}ln\lambda \frac{cF}{{\Phi }^{2}}){k}_{B}T\pi /{d}_{F})}\end{array}$$

where “a” is the parameter related to the emission area, *b* = 6.83(*eV*)^−3/2^(*V*)(*nm*) is the field emission constant, Φ is the surface work function of emitters, T is the nanostructured emitter’s temperature and F is the effective electric field fallen between the cathode and the anode. F can be obtained by the expression of F = βE, in which E is the applied electric field and β is the field enhancement factor. Also, *ν*_*F*_ and *τ*_*F*_ are two correction factors that come from the image potential effects:

2a $${\nu }_{F}=1-\lambda \frac{cF}{{\Phi }^{2}}+\frac{1}{6}\lambda \frac{cF}{{\Phi }^{2}}\,\mathrm{ln}(\lambda \frac{cF}{{\Phi }^{2}})$$

2b $${\tau }_{F}=1+\frac{1}{9}\lambda \frac{cF}{{\Phi }^{2}}-\frac{1}{18}\lambda \frac{cF}{{\Phi }^{2}}\,\mathrm{ln}(\lambda \frac{cF}{{\Phi }^{2}})$$

where $c=\frac{{e}^{3}}{4\pi {\varepsilon }_{0}}$. In addition, Θ_*GSN*_(*T*) describes the finite temperature effect, which can be expressed as:

3 $${\Theta }_{GSN}(T)=\frac{{\tau }_{F}{k}_{B}T\pi /{d}_{F}}{\sin ({\tau }_{F}{k}_{B}T\pi /{d}_{F})}$$

where k_B_ is the Boltzmann constant, ${d}_{F}={({g}_{e}\frac{{\Phi }^{1/2}}{eF})}^{-1}$ and ${g}_{e}=2\sqrt{\frac{2{m}_{e}}{{h}^{2}}}$ are also the constants.

It should be remarked in the generalized SN model that λ is introduced to describe different effective image potential effect from various nanomaterials. When λ=0, the model is simplified as the classical FN model. And the model becomes the idealized SN model when λ=1. Figure 1(a) shows the schematic diagram of quasi one-dimensional nanomaterials during FE measurements. The interface barrier between cathode nanomaterials and vacuum at negatively biased is given in Fig. 1(b) according to the generalized SN model. As found in Fig. 1(b), the image potential factor λ is 1 for metallic nanomaterials, which is easy to understand because the ideal SN model just aims at metal. So the mirror charges should be equal to the surface charges for metallic nanomaterials, which corresponds to the case of λ=1. If nanomaterials belong to semiconductors, the mirror charges will vary with their dielectric constant or electric conductivity, unequal to the surface charges. In this situation, λ ranges from 0 to 1, which should qualitatively reflect the change of the effective mirror charges for different quasi one-dimensional nanomaterials.

In general, it can be believed that the metallic emitters contribute a perfect image potential effect in FE process, namely λ=1, corresponding that the mirror charges should be equal to the surface charges. For semiconductor emitters, the major carriers of the n-type semiconductors are electrons in the bottom of the conduction-band while the major carriers of the p-type semiconductors are holes in the top of the valence-band by doping. In this situation, the mirror charges will vary with their dielectric constant or electric conductivity, unequal to the surface charges. So λ ranges from 0 to 1, which should qualitatively reflect the change of the effective mirror charges for different quasi one-dimensional nanomaterials. In addition, the electrons in the n-type semiconductors emit from the bottom of the conduction band, which should induce the image potential effect a little less than that of metals. In contrast with n-type semiconductors, the contribution of the holes to the image potential in the p-type semiconductors is far less than that of the n-type of semiconductors because the valence-band electrons have to go across the energy-band before they become the free electrons involving in the transportation. Thus, the values of λ also reveal the characteristic of the energy-band structures of nanostructured emitters and reflect the magnitude of the image potential at interface between vacuum and nanomaterials’ surface. Therefore, if we can find out the relationship between the values of λ and the I-V curves of field emission, the conduction-types of nanostructured emitters from field emission can be indeed identified.

On the other hand, the slope of the FN plots has been used to analyze the behaviors of the I-V curves for FN and SN models in our earlier work^18^. Based on Eqs (1)–(3), the slope (S_GSN_) of the FN plots in the generalized SN model can be worked out:

4 $$\begin{array}{rcl}{S}_{GSN} & = & \frac{\partial }{\partial {F}^{-1}}\,\mathrm{ln}\,\frac{{J}_{GSN}}{{F}^{2}}=-{b}_{FN}{\Phi }^{3/2}+\frac{{b}_{FN}{\Phi }^{3/2}}{3}[\frac{11}{2}-\,\mathrm{ln}(\frac{c}{{\Phi }^{2}}\lambda F)]\frac{c}{{\Phi }^{2}}\lambda F\\  &  & +\frac{2c}{9{\Phi }^{2}}\eta \lambda {F}^{2}+F[1-\frac{\lambda c}{18{\Phi }^{2}}\eta F-\frac{G}{\tan \,G}(1-\frac{\lambda c}{18{\Phi }^{2}}\eta F)]\end{array}$$

where ${\rm{G}}=\frac{{\tau }_{F}{k}_{B}T\pi }{{d}_{F}}$ and $\eta =\frac{1-In(\frac{c\lambda F}{{\Phi }^{2}})}{{\tau }_{F}}$. It is found in Eq. (4) that the slopes of FN plots are quite different and depend on the effective electric field and temperature in our generalized SN model. These different behaviors of the slopes of FN plots also provide us another guideline to identify the conduction-type of nanostructured emitters according to the hints of the slope behaviors of the FN plots. Compared with the classical FN or SN models, the slope of the FN plots for the generalized SN model is dependent on the image potential factor λ, which promises us a way to understand the material properties of the nanostructured emitters.

We established the connection between λ and the J-E curves of field emission by combination of the generalized SN model and the slope of FN plots techniques. The basic idea of this scheme is that we use the experimental J-E curves from different nanomaterial emitters to fit the formula in Eq. (1) to obtain the λ values and subsequently calculate the slopes of the FN plots to ascertain the connection between λ and the conduction-types of emitter materials.

For given nanostructured emitters, their work function can be easily measured by UPS or KPFM techniques. So in the fitting process of the J-E curves, only four parameters need to be considered according to Eq. (1), which are respectively a, β, T and λ. In fact, we constrain the a and β within a practical and reasonable region to turn out λ, and then we investigate the slope of FN plots by these parameters and the J-E curves. Different λ values and slopes of FN plots can reveal different nanomaterials’ properties.

The nonlinear fitting method with the Levenberg Marquardt iteration algorithm is utilized in the data-fitting of the J-E curves^19^, which is a standard approach used to solve the nonlinear least squares problems implemented by OriginPro 2016 software (version: Sr2 b9.3.2.303).

## Connection between λ and I-V curves

**Figure 2:**
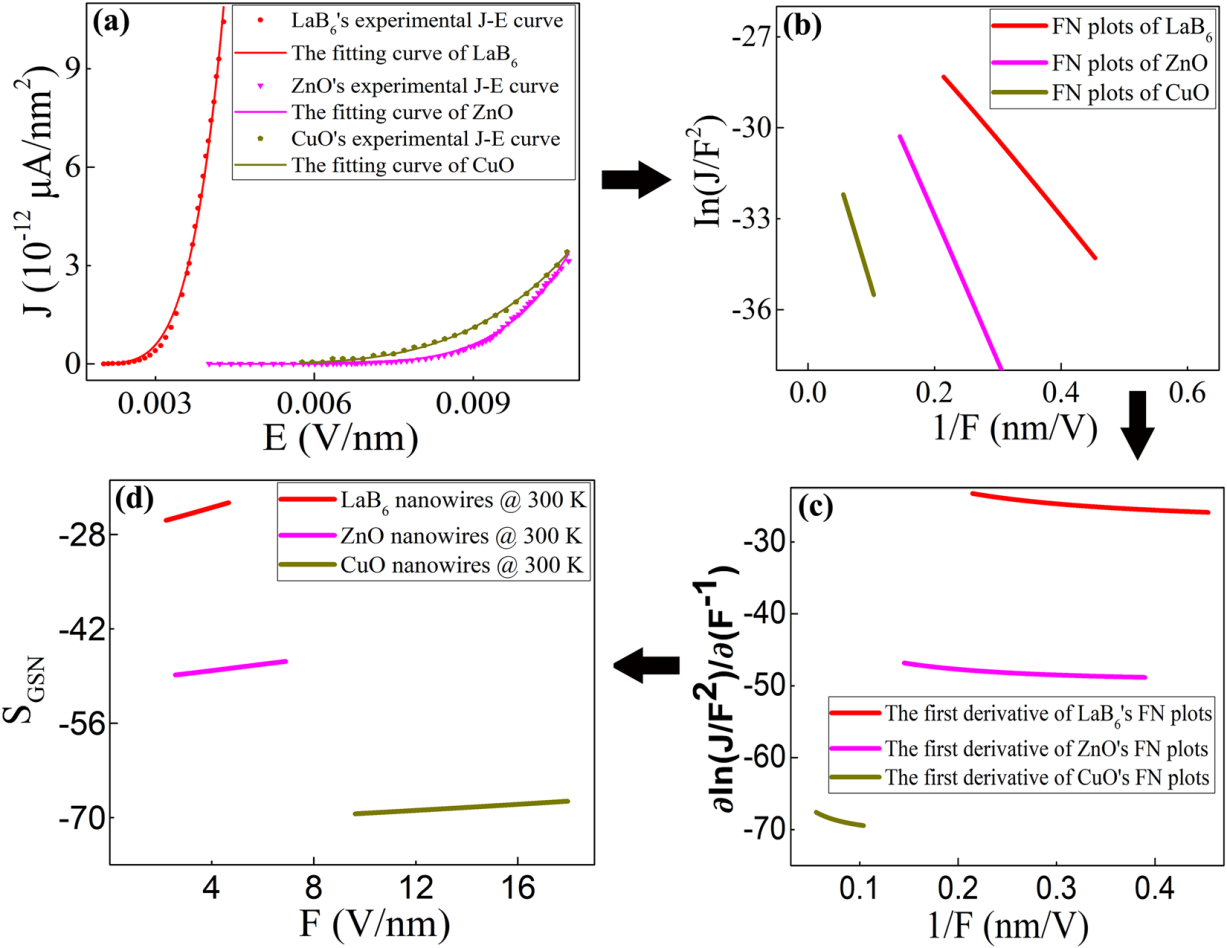
(**a**) The J-E curve-fitting of three typical kinds of metal, n-type and p-type semiconductors and (**b**) their corresponding FN plots. (**c**) The curves of the partial derivative of the FN plots to 1/F. (**d**) The curves of the slopes (S_GSN_) of the FN plots versus the effective electric field (**F**).

Based on our generalized SN model, we fit the experimental J-E curve data from three typical kinds of emitter materials, which are respectively metal (LaB_6_), n-type (ZnO) and p-type (CuO) semiconductors. They are all nanowire materials, whose conduction properties actually have been confirmed by the traditional FET technique. The experimental curves of FE current density of these three types of nanomaterials versus the applied electric field are shown in Fig. 2(a), in which the matching degree of these three fitting curves to the generalized SN equation (Eq. (1)) is over 0.99. Table 1 lists the curve-fitting parameters to further compare their image potential in FE process.

**Table 1:** Nonlinear curve-fitting parameters of the experimental J-E curves for nanomaterial emitters.

Nanomaterials	Nonlinear fitting parameters					
	a (μA)(eV)(V^−2^)	Φ (eV)	β	T (K)	λ	Conduction-type
LaB_6_ nanowires	1.2 × 10^−12^	2.6	1088	300	1	metallic
ZnO nanowires	8.7 × 10^−12^	3.8	639	300	0.65	n-type
CuO nanowires	0.9 × 10^−12^	4.78	1667	300	0.2	p-type
Mo nanocones	7.7 × 10^−12^	4.24	2733	300	1	metallic
SmB_6_ nanowires	8.1 × 10^−13^	4.4	996	300	1	metallic
C nanotubes	2.0 × 10^−11^	4.95	3289	300	0.7	n-type
W_18_O_49_ nanowires	1.8 × 10^−12^	4.5	1860	300	0.7628	n-type
WO_3_ nanowires	5.4 × 10^−11^	4.8	3957	300	0.75	n-type
WO_2_ nanowires	3.5 × 10^−11^	4.6	4745	300	0.8	n-type
AlN nanowires	1.9 × 10^−12^	3.7	1670	300	1.8 × 10^−9^	p-type
B nanowires	1.4 × 10^−13^	4.4	1635	300	0.15	p-type
Individual Si nano-apex	118.8	4.625	2.8	1059	0.86	n-type

Figure 2(b) shows the corresponding FN plots. As observed in Fig. 2(b), the FN plots of these three typical nanowires all exhibit a little deviation from linearity, which suggest that they may obey the generalized SN model. And the curves of the partial derivative of the FN plots to 1/F are shown in Fig. 2(c), in which one can see that these three nanomaterials have different variation tendency. It should attribute to their different effective image potential. It is found in Fig. 2(d) that the curves of the FN plots’ slope (S_GSN_) versus the effective electric field (F) have a different variance tendency for various nanomaterials with different conduction-type. In addition, the slope (S_GSN_) of the FN plots is found to increase linearly with the effective electric field (F) for these three typical nanowires. As seen in Table 1, the image potential factor (λ) is 1 for metallic LaB_6_ nanowires and 0.65 for n-type ZnO nanowires, which is far larger than p-type CuO nanowires (λ = 0.2). It suggests that the image potential factor (λ) may be regarded as the key criterion for identifying the conduction-type of nanomaterials.

**Figure 3:**
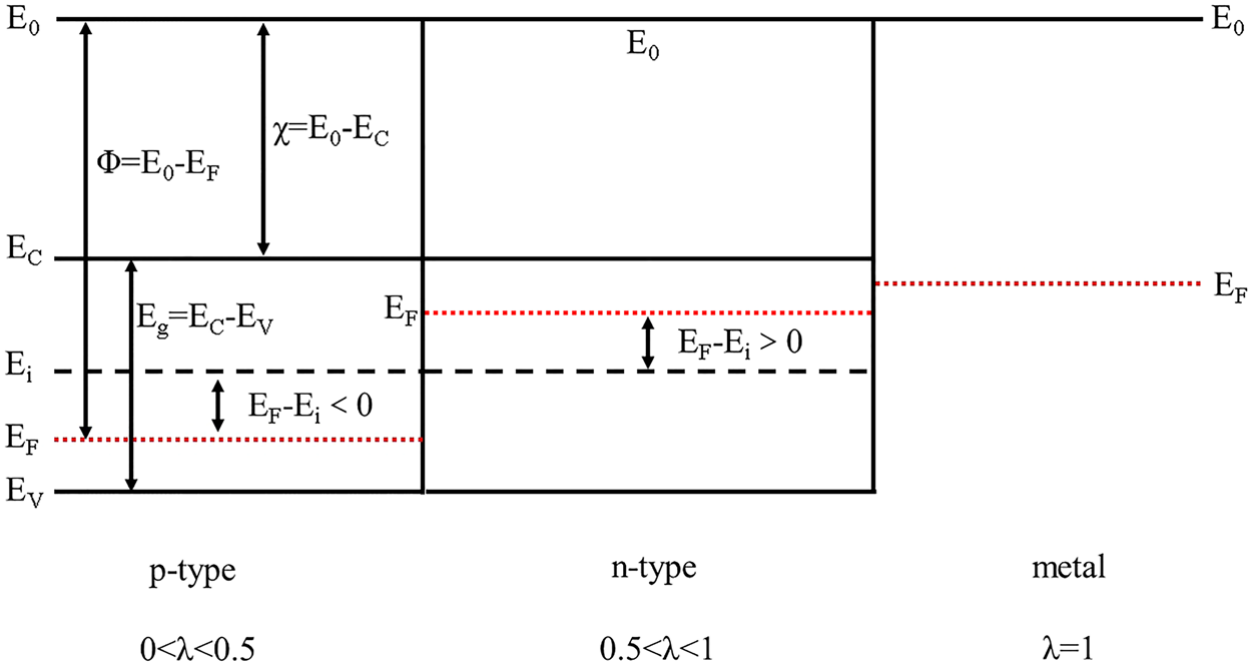
The energy-band diagram of metal, n-type and p-type semiconductors, in which the work function (Φ), electron affinity (χ), bandgap (E_g_) and the positions of the Fermi levels are respectively figured out.

To better comprehend the physical nature of the image potential factor (λ), the energy-band diagram of the nanostructured emitters is shown in Fig. 3. The doping concentration or the relative Fermi level position (E_F_-E_i_) of nanomaterials may be mainly responsible for the magnitude of the image potential factor (λ), in which E_F_ and E_i_ respectively refer to the actual Fermi level and intrinsic Fermi level. As depicted in the above illustrations, λ depends on the surface charges having an intense relationship with the electric conductivity, and thus it should be determined by the major carrier density (n or p) or (E_F_-E_i_).

Based on the semiconductor physics, the electric conductivity (σ) of nanomaterials can be worked out by the equation of *σ* = *nqμ* or *σ* = *pqμ* ^20^. In these expressions, μ is the mobility of major carriers in semiconductors and q stands for elementary charge. According to the classical band theory, E_F_ is just above E_i_ for n-type semiconductors whereas E_F_ is lower than E_i_ for p-type semiconductors, as seen in Fig. 3. The major carrier density (n or p) can be deduced by the Fermi distribution law:

5 $$n={n}_{i}ex{p}^{(\frac{{E}_{F}-{E}_{i}}{{k}_{B}T})}$$

where k_B_ is the Boltzmann’s constant, T is the temperature and n_i_ is the intrinsic carrier density. Based on the semiconductor physics, n_i_ can be expressed as:

6 $${n}_{i}=4.82\times {10}^{15}{(\frac{{{m}_{n}}^{\ast }{{m}_{p}}^{\ast }}{{{m}_{0}}^{2}})}^{3/4}{T}^{3/2}ex{p}^{(-\frac{{E}_{g}}{2{k}_{B}T})}$$

where E_g_ is the bandgap of nanomaterials, m_0_ is the electron mass, m_n_^*^ and m_p_^*^ are respectively the effective masses of electron and hole. Substituting Eqs (5) and (6) into the above equation on the electric conductivity σ, we can obtain:

7 $${E}_{F}-{E}_{i}=\frac{{E}_{g}}{2}+{k}_{B}T\,\mathrm{ln}[\frac{2\times {10}^{-16}\sigma }{q\mu {T}^{3/2}}{(\frac{{{m}_{n}}^{\ast }{{m}_{p}}^{\ast }}{{{m}_{0}}^{2}})}^{-3/4}]$$

In general, the energy-band structure, the carrier density, the electric conductivity and temperature may modify the image potential, and thus take effect on the J-E curves of field emission. Although the effective image potential factor λ is introduced to describe these nanomaterials in our generalized SN model, the connection between λ and the conduction-type of emitters can be preferred that (1) λ = 1 for metals; (2) 0.5 <λ < 1 for n-type semiconductors; (3) 0 < λ < 0.5 for p-type semiconductors. Therefore, the relationship between the J-E curves and the conduction-type of emitter in FE process can be easily revealed by this simple way instead of fabricating complicated FET nanodevices. Moreover, this connection should have another application, which is to comprehend the FE mechanism and analyze the doping degree of the nanomaterials when we know what material the emitters belong to.

Based on the above-mentioned analysis, our identification method of the conduction-type of nanomaterials can be summarized into three standard steps:

1. Fitting the J-E data based on the generalized SN model presented in the above section;
2. Calculating the slope of FN plots based on the parameters obtained by the fitting data in the first step;
3. Obtaining the λ values and drawing the curve of the slope (S_GSN_) of FN plots to λ for different quasi one-dimensional nanomaterials.

**Figure 4:**
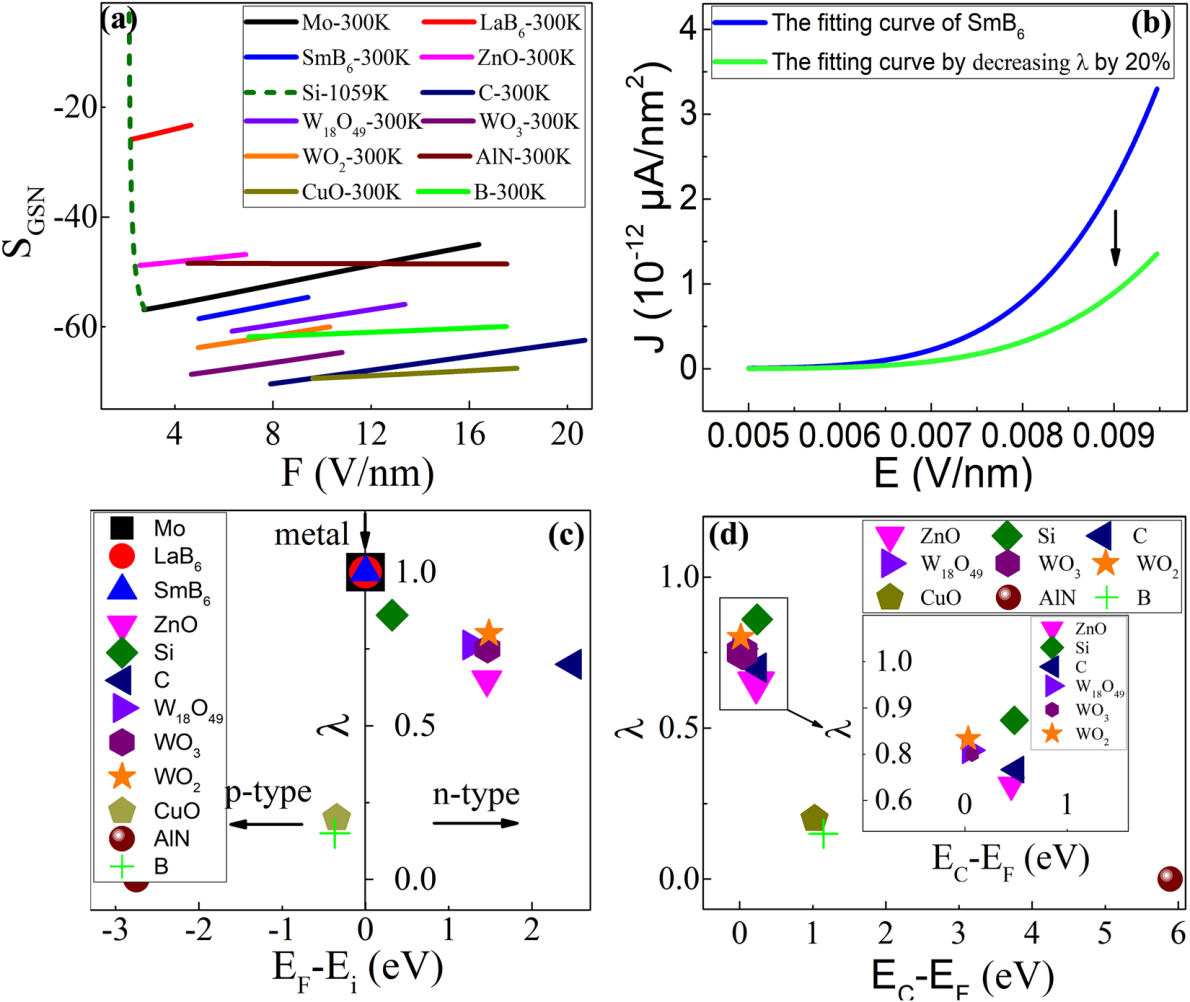
(**a**) The slopes of FN plots versus F for various nanomaterial emitters; (**b**) The fitting J-E curves of SmB_6_ nanowires with different λ values for comparison; (**c**,**d**) The curves of λ versus (E_F_-E_i_) and (E_C_-E_F_), respectively.

Furthermore, we investigate a group of samples to testify the as-obtained λ criterion based on this method. The samples include Mo nanocones, SmB_6_ nanowires, individual Si nano-apex, C nanotubes, W_18_O_49_ nanowires, WO_3_ nanowires, WO_2_ nanowires, AlN nanowires and B nanowires. All of them are prepared by our group, and their fabrication techniques can be found in the Supporting information. The nonlinear curve-fitting and calculation process of these experimental J-E data is just the same as the aforementioned three quasi one-dimensional nanomaterials (LaB_6_ nanowires, ZnO nanowires and CuO nanowires), as found in Figs S1 and S2 in Supporting Information. Before the measurements, the conduction-type of these nine kinds of nanomaterials remain unknown to us. The S_GSN_-F curves of these nine quasi one-dimensional nanomaterials are shown in Fig. S2(c). As observed in Fig. 4(a), the slopes (S_GSN_) of FN plots versus the electric field (F) are found to be different for these nanomaterials. Based on the generalized SN model, the parameters can be solved, as summarized in Table 1. According to the criterion of the aforementioned image potential factor (λ), it can be concluded that Mo nanocones and SmB_6_ nanowires are metallic because the λ values are equal to 1; Individual Si nano-apex, C nanotubes, W_18_O_49_ nanowires, WO_3_ nanowires and WO_2_ nanowires can be ascertained to be n-type semiconductors in that their λ value ranges from 0.5 to 1; Likewise, AlN nanowires and B nanowires should belong to p-type semiconductors since their λ values locate at the range from 0 to 0.5. The conduction-type of these nine quasi one-dimensional nanomaterials differentiated by the image potential method is in good consistent with those by traditional FET method, which proves that our method is accurate and suitable for determination of the conduction-type of quasi one-dimensional nanomaterials.

It is also found that there are three kinds of behaviors of the slopes (S_GSN_) of FN plots with F. One is seen to remain almost unchanged for AlN, B and CuO nanowires; The second is observed to increase linearly with F for Mo nanocones, SmB_6_ nanowires, C nanotubes, W_18_O_49_ nanowires, WO_3_ nanowires and WO_2_ nanowires. The third exhibits a very sharp negative slope with F for individual Si nano-apex. It should be noted that all the temperatures of nanomaterials during FE process are 300 K except individual Si nano-apex (T = 1059 K), which can be illustrated as follows. The FE properties of nanowire films can be believed to be room-temperature measurements by transparent anode way because the nanowires contact well with the substrate and can rapidly conduct the heat to the external circumstance, in which the Joule heat at nanowire films can be neglected. But for the individual Si nano-apex prepared by EBL technique, a native oxide layer usually formed on the surface of Si substrate, which inevitably leads to a large interface resistance between individual Si nano-apex and Si substrate. This high interface resistance produces Joule heat in FE process without a nice channel to timely dissipate the heat to external circumstance during *in-situ* microprobe measurements. Under this circumstance, it results that the temperature of an individual Si nano-apex reaches over 1000 K, which coincides with our experimental observations very well^21^. Therefore, it is reasonable that the fitting temperature T is adopted to 1059 K for individual Si nano-apex. Moreover, it can be identified that Si nano-apex belongs to the n-type semiconductor because S_GSN_ decreases with the increase of temperature based on Eq. (4). The variance tendency of S_GSN_ to F is totally different at 300 K and 800 K for the original SN model as shown in ref.^18^. Moreover, it provides another powerful proof for differentiating the conduction-type of quasi one-dimensional materials. It is noted that the variable behaviors of S_GSN_ with the temperature also give us a signal to estimate the working temperature of different conduction-type nanostructured emitters.

The image potential may affect the FE properties of quasi one-dimensional nanomaterials, so here the J-E curves of SmB_6_ nanomaterials are provided in Fig. 4(b) as an example. If λ is decreased by 20% in the curve-fitting process, J will accordingly decrease by 61.4% in comparison with that before change at the same turn-on field of 6.5 V/μm. Therefore, it suggests that the image potential should have a strong impact on the FE behaviors of quasi one-dimensional nanomaterials, which is also beneficial to identify the conduction types by the image potential method because λ significantly differs with their conduction-type.

We endeavor to explore what is the determinant factor for the image potential factor (λ), and the curve of λ to the relative Fermi level position (E_F_-E_i_) is first given in Fig. 4(c). The electric conductivity of nanomaterials measured by the *in-situ* microprobe technique were listed in Table 2 ^21–26^, and their mobility and bandgap adopt the averaged value of their corresponding bulk counterparts in recent studies^15,24,27–33^. Combined Eq. (7) with the measurement results, the difference (E_F_-E_i_) between E_F_ and E_i_ can be solved for all nanomaterials, as summarized in Table 2. One can see that (E_F_-E_i_) is positive when λ ranges from 0.5 to 1, which reveals these nanomaterials (ZnO nanowires, individual Si nano-apex, C nanotubes, W_18_O_49_ nanowires, WO_3_ nanowires and WO_2_ nanowires) should be n-type semiconductors. These results concide with our aboved-mentioned conclusions very well. Simultaneously, (E_F_-E_i_) appears to be negative if λ is located in the range from 0 to 0.5 for all p-type semiconductors, which is also in good agreement with the given criterion. Therefore, it proves that our way by regarding the λ value as the criterion should be reasonable and accurate for idenficiation of the conduction-type of nanomaterials.

**Table 2:** Physical parameters of nanomaterial emitters.

Nanomaterials	Physical parameters						
	Bandgap (eV)	Conductivity (Ω^−1^cm^−1^)	Mobility (cm^2^V^−1^s^−1^)	E_F_–E_i_ (eV)	E_C_-E_F_ (eV)	λ	Conduction-type
LaB_6_ nanowires	0	1.43 × 10^4^	32	0	—	1	metallic
ZnO nanowires	3.37	7.13 × 10^−2^	200	1.458	0.227	0.65	n-type
CuO nanowires	1.36	3.92 × 10^−4^	70	−0.345	1.025	0.2	p-type
Mo nanocones	0	3.44 × 10^4^	5	0	—	1	metallic
SmB_6_ nanowires	0	3.8 × 10^3^	20	0	—	1	metallic
C nanotubes	5.47	3.24	2000	2.485	0.25	0.7	n-type
W_18_O_49_ nanowires	2.6	14.3	16.2	1.275	0.025	0.7628	n-type
WO_3_ nanowires	3	9.7	16.2	1.465	0.035	0.75	n-type
WO_2_ nanowires	3	20.6	16.2	1.484	0.016	0.8	n-type
AlN nanowires	6.28	2.7 × 10^−4^	14	−2.750	5.89	1.8 × 10^−9^	p-type
B nanowires	1.56	1.66 × 10^−2^	4000	−0.368	1.148	0.15	p-type
Individual Si nano-apex	1.124	200	1450	0.320	0.242	0.86	n-type

It is clearly seen in Fig. 4(c) that the image potential factor (λ) shows two quite different changing tendency for different conduction-type of nanomaterials. For n-type semiconductors, λ will increase with (E_F_-E_i_) and gradually moves close to 1 (ideal metal plane), in which (E_F_-E_i_) actually reflects the doping degree of nanomaterials. But for p-type semiconductors, λ will decrease with the increase of (E_F_-E_i_) and even be close to 0 for heavily-doped p-type semiconductors or wide-bandgap semiconductors with quite a few conduction-band electrons. The possible explanations are given as follows. Theoretically speaking, the image potential (V_image_) can be written as ${V}_{image}=-\frac{{e}^{2}}{16\pi {\varepsilon }_{0}x}$, in which “e” represents the mirror charges at vacuum side and x stands for the distance between surface charges and cathode surface. As a result, the magnitude of the image potential (V_image_) depends on the mirror charges at vacuum side. As explained in aforementioned descriptions, the intrinsic nature of λ directly reflects the image potential (V_image_) at interface as well as reveals the contribution of the effective image charges to V_image_, which has an intense relationship with the conduction-type of nanomaterials. So it can be understood that V_image_ should be determined by (E_F_-E_i_) or (E_C_-E_F_) as found in Fig. 4, which also conforms to Eqs (5)–(7). In this situation, the λ values increase with the increase of (E_F_-E_i_) for the same n-type semiconductors and close to 1 for ideal metal, which implies the behaviors of n-type semiconductors become more and more closer to those of metal with the increase of λ. Unlike n-type semiconductors, the hole density of valence-band turns larger with the increase of (E_F_-E_i_) for p-type semiconductors. Accordingly, the conduction-band electron density decreases with the increase of (E_F_-E_i_), which suggests that p-type semiconductors gradually deviate far way from ideal metal. Consequently, λ turns smaller and smaller with the increase of (E_F_-E_i_) and is even close to zero (tiny conduction-band electrons) for p-type semiconductors, which suggests that the doping degree of p-type semiconductors increases with the decrease of λ. So it is reasonable that there exists different variance tendency of λ for n-type and p-type semiconductors with (E_F_-E_i_). Most of all, qualitatively identification of the doping degree becomes possible by comparing their λ values for the same semiconductor nanomaterials.

The relationship between λ and the difference (E_C_-E_F_) between E_C_ and E_F_ is also discussed in Fig. 4(d). One can see in Fig. 4(d) that λ will increase with the decrease of (E_C_-E_F_) for all semiconductor nanomaterials. The possible explanations are given as follows. Theoretically speaking, the conduction-band electron concentration (n) of semiconductor can be written as $n={N}_{C}ex{p}^{(-\frac{{E}_{C}-{E}_{F}}{KT})}$, in which N_C_ represents the effective density of states (DOS) of the conduction-band. Therefore, the conduction-band electron density (n) will turn larger with the decrease of (E_C_-E_F_) based on the aforementioned equation for conduction-band electron density. Under this circumstance, the image potential V_image_ will accordingly increase with (E_C_-E_F_), which leads to the increase of λ value. So it is well understood that λ should exhibit the identical variance tendency whether for n- or p-type quasi one-dimensional nanomaterials. It comes to a conclusion that the conduction-band electron density (n) of quasi one-dimensional nanomaterials should be the determinant factor for λ in comparison with other parameters because it overall considers the contribution of the bandgap (E_g_) and the doping degree of the semiconductor. This method may be expanded to the identification of the conduction-type or the doping degree of other two-dimensional nanostructures or bulk materials, and the relevant researches are still undergoing.

## Conclusions

In summary, we have developed a simple FE technique to fast identify the conduction types of nanomaterials. Furthermore, we established the generalized Schottky-Nordheim (SN) model and introduce the image potential factor (λ) to describe the effective image potential in FE process. By our method, the relationship between λ and the energy-band structure of the nanomaterial emitters can be obtained: (a) λ = 1: metal; (b) 0.5 < λ < 1: n-type semiconductor; (c) 0 < λ < 0.5: p-type semiconductor. This criterion provides us a simple and effective way to identify the conduction types of the nanomaterial emitters and understand the physical mechanism of field emission. We test a group of nanomaterial emitters, and all results agree with both our theoretical predictions and other measurement results by FET technique. In contrast with the conventional hot and cold probe or Hall effect methods, our method is not only more convenient and effective but also can qualitatively determine the doping degree for the same semiconductor nanomaterials. Therefore, our method based on the FE technique should be a powerful tool to identify the conduction-type of nanomaterials and may shed new light on other two- or three-dimensional materials.

## Experimental

### Synthesis methods of twelve kinds of quasi one-dimensional nanomaterials

In our work, twelve kinds of quasi one-dimensional nanomaterials (Mo, LaB_6_, SmB_6_, ZnO, Si, C, W_18_O_49_, WO_3_, WO_2_, CuO, AlN and B) prepared by our laboratory were randomly chosen as the research goals. Nearly all of these nanomaterials were grown by chemical vapor deposition method^22,34–36^ except Mo nanocones^25^ (physical vapor deposition) and Si nano-apex^21^ (electron beam lithography). Moreover, these one-dimensional nanomaterials were found to have identical morphology and uniformly distributed all over the substrate, which can eliminate the effect of nonuniformity on the physical property measurements and further assures the accuracy of the experimental results.

### Physical property measurements of quasi one-dimensional nanomaterials

FE measurements on quasi one-dimensional nanomaterial thin film were carried out in our field emission analysis system, and the measurements on individual nanomaterials were performed in our modified SEM system, as described in our recent works^23^. The schematic diagram of nanomaterials during FE measurements is provided in Fig. 1(a). The base pressure of the measurement chamber is lower than 5 × 10^−5^ Pa, and the distance between cathode and anode ranges from 100 to 500 μm except individual Si nano-apex (100 nm). UPS (Thermo Fisher Scientific, ESCALAB 250) and Kelvin probe force microscopy (KPFM, Omicron VT-AFM) techniques were respectively used to measure the work function of ZnO^37^, CuO^38^ and B^39^ nanowires while other one-dimensional nanomaterials adopted the work function of their corresponding bulk counterparts^24,35,39–41^. Their electric conductivity was also obtained by our modified *in-situ* microprobe measurement technique.

## Electronic supplementary material

supporting informations
