# Supplementary material for: Fast identification of the conduction-type of nanomaterials by field emission technique
Source: Sci Rep. 2017 Oct 12;7:13057. doi: 10.1038/s41598-017-12741-5 (PMC5638822; doi:10.1038/s41598-017-12741-5)
Supplement: Supplementary file 1 — supporting informations [file 41598_2017_12741_MOESM1_ESM.pdf]

Supporting Information

Fast identification of the conduction-type of nanomaterials by field emission technique

Xun Yang[ǂ], Haibo Gan[ǂ], Yan Tian, Luxi Peng, Ningsheng Xu, Jun Chen, Huanjun Chen, Shaozhi Deng[*], Shi-Dong Liang[*] and Fei Liu[*]

State Key Laboratory of Optoelectronic Materials and Technologies,

Guangdong Province Key Laboratory of Display Material and Technology,

School of Electronics and Information Technology, Sun Yat-sen University,

Guangzhou 510275, P. R. China

[*]*E-mail: stsdsz@mail.sysu.edu.cn, stslsd@mail.sysu.edu.cn, liufei@mail.sysu.edu.cn*

[ǂ]*These authors contribute equally to this work*

Table of Contents

Table of contents 2

The nonlinear curve-fitting procedure for other nine nanomaterials 3

The following procedures based on the fitting data of J-E curves 4

Figure captions of Supporting Information.. 5

The nonlinear fitting curves of J-E data for other nine nanomaterials 6

Data processing based on curve-fitting results 7

The nonlinear curve-fitting procedure for other nine nanomaterials

As shown in Fig. S1, Eq. (1) was adopted to fit the experimental J-E curves of other nine nanomaterials, similar with the fitting process of the aforementioned three nanomaterials (LaB_6 nanowires, ZnO nanowires and CuO nanowires). It is found that the matching degree of nearly all the experimental J-E curves to the generalized SN equation is over 0.99 for these nine quasi one-dimensional nanomaterials (Mo nanocones, SmB_6 nanowires, individual Si nano-apex, C nanotubes, $W_{18}O_{49}$ nanowires, WO_3 nanowires, WO_2 nanowires, AlN nanowires and B nanowires). Therefore, it suggests that all the experimental J-E data points of these nine nanomaterials conform to the generalized SN model very well.

The following procedures based on the fitting data of J-E curves

Fig. S2(a) gives the corresponding FN plots of other nine nanomaterials. As seen in Fig. S2(a), the FN plots of all these nanomaterials exhibit more or less deviation from the linearity, suggesting that they should obey the generalized SN model. And the curve of $\frac{\partial}{\partial F^{-1}} ln \frac{J_{S(F)N}}{F^2}$ versus 1/F are shown in Fig. S2(b) based on the above results. As shown in Fig. S2(b), one can see that these quasi-one-dimensional nanomaterials have different variation tendency, which should attribute to their different effective image potential (V_{image}). Therefore, it comes to a conclusion that our criterion by the image potential factor (λ) should be reasonable for detecting the conduction-type of these nanomaterials, which can be widely spread for studying other nanomaterials.

Figure captions

Figure S1. The nonlinear fitting curves of the J-E data for other nine quasi one-dimensional nanomaterials based on Eq. (1). (a) Mo nanocones. (b) SmB_6 nanowires. (c) individual Si nano-apex. (d) C nanotubes. (e) WO_3 nanowires. (f) WO_2 nanowires. (g) $W_{18}O_{49}$ nanowires. (h) AlN nanowires. (i) B nanowires.

Figure S2. Data processing of other nine nanomaterials (Mo, SmB_6, Si, C, WO_3, WO_2, $W_{18}O_{49}$, AlN, B). (a) Their corresponding FN plots based on the fitting curve obtained in the first step. (b) The curves of the partial derivative of the FN plots to 1/F. (c) The curves of the slopes (S_{GSN}) of the FN plots versus the effective electric field (F).

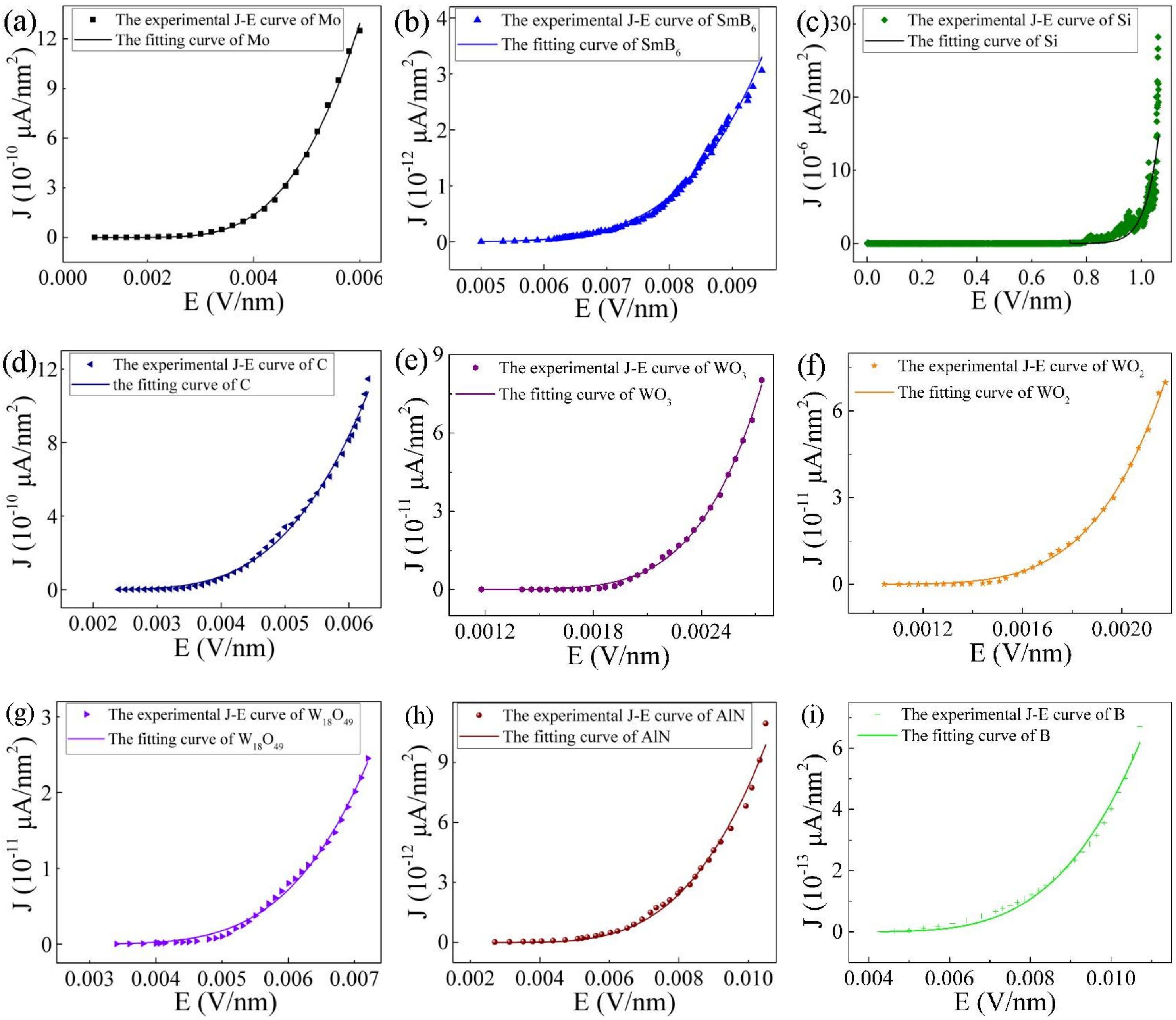
(a)
The experimental J-E curve of Mo
The fitting curve of Mo
J (10^{-10} μA/nm^2)
E (V/nm)
(b)
The experimental J-E curve of SmB$_6$
The fitting curve of SmB$_6$
J (10^{-12} μA/nm^2)
E (V/nm)
(c)
The experimental J-E curve of Si
The fitting curve of Si
J (10^{-6} μA/nm^2)
E (V/nm)
(d)
The experimental J-E curve of C
the fitting curve of C
J (10^{-10} μA/nm^2)
E (V/nm)
(e)
The experimental J-E curve of WO$_3$
The fitting curve of WO$_3$
J (10^{-11} μA/nm^2)
E (V/nm)
(f)
The experimental J-E curve of WO$_2$
The fitting curve of WO$_2$
J (10^{-11} μA/nm^2)
E (V/nm)
(g)
The experimental J-E curve of W$_{18}$O$_{49}$
The fitting curve of W$_{18}$O$_{49}$
J (10^{-11} μA/nm^2)
E (V/nm)
(h)
The experimental J-E curve of AlN
The fitting curve of AlN
J (10^{-12} μA/nm^2)
E (V/nm)
(i)
The experimental J-E curve of B
The fitting curve of B
J (10^{-13} μA/nm^2)
E (V/nm)

Figure S1.

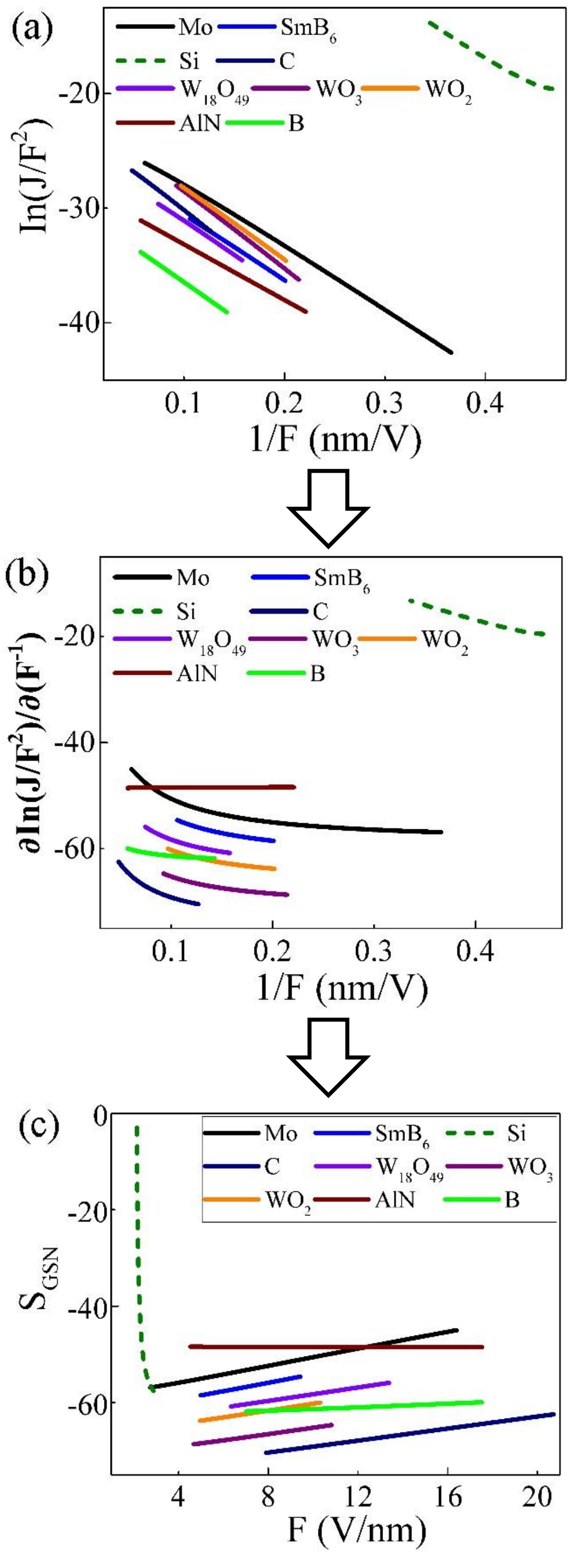
(a)
Mo
SmB6
Si
C
W18O49
WO3
WO2
AlN
B
ln(J/F2)
-20
-30
-40
0.1
0.2
0.3
0.4
1/F (nm/V)
(b)
Mo
SmB6
Si
C
W18O49
WO3
WO2
AlN
B
∂ln(J/F2)/∂(F-1)
-20
-40
-60
0.1
0.2
0.3
0.4
1/F (nm/V)
(c)
Mo
SmB6
Si
C
W18O49
WO3
WO2
AlN
B
SGSN
0
-20
-40
-60
4
8
12
16
20
F (V/nm)

Figure S2.
